# Gluteal Flap Reconstruction Following Complex Rectal Cancer Surgery: A Large Consecutive Series of Perineal Wounds Exploring Risk Factors for Complications

**DOI:** 10.1002/jso.70113

**Published:** 2025-11-05

**Authors:** Laura E. Gould, Edward. T. Pring, Ioanna Drami, Joannis Constantinides, Nicola Hodges, Colin W. Steele, Campbell S.D. Roxburgh, Elaine M. Burns, John T. Jenkins

**Affiliations:** ^1^ Complex Cancer Clinic St Mark′s‐The National Bowel Hospital Harrow London UK; ^2^ St Mark′s Academic Institute St Mark′s‐The National Bowel Hospital Harrow London UK; ^3^ School of Cancer Sciences, College of Medical, Veterinary & Life Sciences University of Glasgow Glasgow UK; ^4^ Department of Surgery and Cancer Imperial College London UK; ^5^ University Department of Surgery Glasgow Royal Infirmary Glasgow UK

## Abstract

**Aim:**

To determine whether high complexity pelvic exenterations alter perineal wound morbidity and to assess risk factors for perineal flap complications following complex rectal cancer surgery.

**Methods:**

A retrospective analysis of consecutive adults undergoing complex rectal cancer resections with immediate gluteal flap perineal reconstruction between January 2013‐July 2021 at a tertiary referral centre. Conventional complex cancer resections were compared with “high complexity” exenterations, including en bloc sacrectomy and extended lateral pelvic side wall excision. Primary outcomes were short‐term (wound infection, necrosis, dehiscence) and long‐term (sinus, fistula, hernia) perineal flap complications.

**Results:**

We identified 194 patients (median 56 years, 60% male) with gluteal flap reconstructions; 163 (84%) for advanced or recurrent rectal cancer. Gluteal artery perforator flaps were predominantly used (176, 92%). Wound infections were more common in the conventional group (23.2% vs. 6.3%, *p* = 0.001), but no other differences in complications were observed between groups. Obesity (HR 2.70, 95% CI 1.22–5.97, *p* = 0.014) and total pelvic exenteration (HR 2.13, 95% CI 1.07–4.23, *p* = 0.031) were associated with short‐term complications. Age over 65 years predicted readmission/reoperation (HR 2.66, 95%CI 1.07–6.6, *p* = 0.040). Ureteric/ileal conduit leaks were associated with long‐term complications (HR 3.37, 95% CI 1.21–9.34, *p* = 0.024). No flap losses occurred.

**Conclusion:**

Gluteal fasciocutaneous perforator flaps provide reliable perineal reconstruction after complex rectal cancer surgery. The extent of surgery and resulting defect size did not significantly influence perineal wound complication rates.

## Summary

This large consecutive series (*n* = 194) evaluates predominantly gluteal fasciocutaneous perforator flaps (*n* = 163) for perineal reconstruction after complex rectal cancer surgery, comparing conventional exenterations with “high‐complexity” resections (en bloc sacrectomy and/or ELSiE). Overall, perineal flap complication rates were similar between groups, though wound infection was higher after conventional surgery; independent risk factors included obesity (BMI > 30) and total pelvic exenteration for short‐term complications, age > 65 for readmission/reoperation, and ureteric/ileal conduit leaks for long‐term problems—importantly, there were no flap losses. These findings support gluteal perforator flaps as reliable reconstructions across defect sizes and highlight modifiable and procedural risk factors to optimise perioperative management.

- **What Does This Paper Add to the Literature?′**
  ∘ This study demonstrates that gluteal fasciocutaneous perforator flaps provide reliable perineal reconstruction after complex rectal cancer surgery, with similar complication rates regardless of defect size. It identifies specific risk factors for complications, guiding patient selection and perioperative management that may optimise outcomes.

## 1 Introduction

Advances in surgical techniques have transformed the management of locally advanced and recurrent rectal cancer, offering curative resection for patients previously deemed inoperable. Infiltration of the sacrum and pelvic side wall are no longer contraindications to curative resection, with 3‐year survival rates of 48%–56% now achievable in these challenging cases [1, 2, 3]. However, this aggressive surgical approach carries significant morbidity, with 40% of patients experiencing major postoperative complications [2, 3, 4].

Perineal wound complications remain a substantial contributor to postoperative morbidity. Pelvic exenteration for rectal cancer often produces large perineal defects not amenable to primary closure, necessitating collaboration with plastic surgeons to achieve wound coverage and closure, frequently requiring flap reconstruction. Following pelvic exenteration, reconstruction aims to achieve primary wound closure and obliterate the “dead space” by introducing well‐vascularized tissue to previously irradiated areas [5].

Various flaps have been described for perineal closure, including musculocutaneous flaps from the abdomen (vertical rectus abdominis myocutaneous ‐ VRAM) and thigh (gracilis), fasciocutaneous flaps (inferior ‐ IGAP and superior ‐ SGAP gluteal artery perforator, anterolateral thigh, posterior thigh), and regional fasciocutaneous flaps (e.g., Lotus pedal or Singapore) [5, 6]. Flap use following exenteration surgery varies from 22.6% to 40% in published series [2, 3, 7]. While various algorithms have been proposed for flap selection, the optimal management of perineal defects remains unclear [8].

Perineal wound complications may be associated with underlying patient or surgical factors and result in significant sequelae, including increased length of postoperative stay, delays in adjuvant therapy, and substantial healthcare costs [9]. Understanding risk factors for complications is essential for optimizing patient outcomes.

This study aims to present our long‐term experience using primarily gluteal flap reconstruction in patients undergoing resection of locally advanced and recurrent rectal cancer in a dedicated tertiary referral unit. We sought to identify risk factors associated with postoperative perineal reconstruction complications and to determine whether high‐complexity exenteration surgery contributes to increased perineal morbidity.

## 2 Methods

### 2.1 Study Design and Population

We conducted a retrospective analysis of prospectively collected data from all consecutive complex rectal cancer operations undertaken at St Mark′s The National Bowel Hospital, London, UK, from January 2013 to July 2021. All cases involving anterior, posterior, or total pelvic exenteration for malignancy, or extended extralevator abdominoperineal excision (ELAPE) were included. Palliative or abandoned operations were excluded. Data were compiled or revalidated using electronic records, clinic letters, specialist multidisciplinary team (MDT) reports, and laboratory systems. The study followed STROBE reporting guidelines for cohort studies.

### 2.2 Data Collection

Demographic data included age, gender, American Society of Anesthesiologists (ASA) grade, comorbidities, body mass index (BMI), smoking status, malignancy subtype, and radiotherapy history (both short and long course radiotherapy included). Data were validated with Cancer Registry data (Office for Data Release, Public Health England).

Operative records were reviewed and coded according to the extent and complexity of the procedure, flap type, bony resection, and whether vascular or urological reconstruction was required. “High complexity” operations were defined as those including extended lateral pelvic side wall excisions (ELSiE), en bloc sacrectomy, high sacrectomy, or high subcortical sacrectomy (HiSS), as defined in the ACPGBI/UKPEN Pelvic Exenteration Lexicon [10, 11, 12, 13]. This cohort was compared with those undergoing conventional pelvic exenteration operations without en bloc resection of bony structures, ligaments, or neurovascular components of the pelvic sidewall(s).

### 2.3 Outcomes

Primary outcomes included postoperative perineal flap‐related complications: short‐term (wound infection, flap necrosis, wound dehiscence) and long‐term (sinus, fistula, perineal hernia). Wound infection was defined as a clinically infected wound, with or without positive microbiology, requiring antimicrobial therapy. Wound dehiscence was defined as any significant separation of the wound requiring packing or VAC therapy.

Secondary outcomes included postoperative length of stay, 30‐day mortality, and complications categorized by Clavien‐Dindo classification [14], with grade ≥ 3 considered major. We recorded return to theatre for perineal wound issues within 90 days, readmissions, and wound revisions. A major flap complication was defined as return to theatre within 90 days or readmission within 30 days due to any wound‐related issue [15].

### 2.4 Surgical Technique

All patients were discussed at a specialist MDT, where the extent of tumour infiltration was assessed using MRI. The operative plan was based on the maximum disease extent demonstrated on preoperative imaging. A systematic approach determined the boundaries of resection with consideration given to urological, bony, or vascular resection requirements. The flap type used was based on defect size and available blood supply (dependent on the extent of pelvic side wall and sacral resection).

Postoperatively, patients were nursed on their side for 7 days, with standing allowed from day one. If the flap was healthy, sitting was gradually introduced from day seven, increasing by 5‐min increments daily until day ten when patients could sit as tolerated on an appropriate pressure‐relieving cushion. Review by the plastic surgery team and Tissue Viability nursing staff assessed the need for dressings or vacuum‐assisted closure (VAC) therapy in cases of superficial wound dehiscence [16].

### 2.5 Ethical Approval

Approval was obtained for use of the database from the NHS Health Research Authority with ethical approval from the South East London NHS Research Ethics Committee (reference [12]/LO/1556).

### 2.6 Statistical Analysis

Continuous variables are presented with median and interquartile range (IQR), and categorical data with frequency and percentage. The Mann–Whitney U‐test was used for continuous data and Fisher′s Exact test or Pearson′s chi‐square test for categorical data. Multivariate analysis was performed by binary logistic regression using only variables statistically significant on univariate analysis. A *p*‐value < 0.05 was considered significant. Kaplan‐Meier survival plots were performed and differences between groups assessed using a log rank test. Data were analysed using *SPSS* software (version 24) (*IBM, Armonk, New York, USA)*.

## 3 Results

From January 2013 to July 2021, 453 complex rectal cancer operations were performed; 194 patients underwent immediate perineal flap reconstruction. Most patients were men (60.5%) with a median age of 56 years (IQR 48‐66). Due to the magnitude of surgery, patients had few comorbidities with 80% (*n* = 155) being ASA 2 and only 20% (*n* = 39) ASA 3. [Table 1.]

**Table 1:** Patient demographics and operative details.

	Conventional Complex resection *n* = 82 [%]	High Complexity resection *n* = 112 [%]	*P* value		Conventional Complex resection *n* = 82 [%]	High Complexity resection *n* = 112 [%]	*P* value
**Age, Median years (IQR)**	56 (50–67.25)	57 (47–65)	0.626	**Surgical resection**			
**Sex**							
Male	46 (56.1)	71 (63.4)	0.373	Extended APER	20 (24.4)	0	**< 0.001**
Female	36 (43.9)	41 (36.6)		Anterior pelvic exenteration	1 (1.2)	1 (0.9)	1.00
**Comorbidity**				Posterior pelvic exenteration	34 (41.5)	42 (37.5)	0.655
IHD	4 (4.9)	4 (3.6)	0.651	Total pelvic exenteration	27 (32.9)	69 (61.6)	**< 0.001**
Type 2 diabetes	3 (3.7)	8 (7.1)	0.361	Oncovascular resection	0	8 (7.1)	**< 0.001**
HTN	14 (17.1)	12 (10.7)	0.209	Ileal conduit	28 (34.1)	70 (62.5)	**< 0.001**
IBD	15 (18.3)	9 (8.0)	**0.046**	Boari or ureteric reimplantation	1 (1.2)	8 (7.1)	0.081
Current smoker	9 (11)	3 (2.7)	**0.031**	**High Complexity resections**			
**BMI (*n*=157)**				Sacrectomy/HiSS	0	89 (79.5)	
Median, IQR	25.9 (23.4–30.6)	25.0 (22.5–30.4)	0.458	ELSiE	0	97 (86.6)	
Over 30 kg/m^2^	17 (20.7)	20 (17.9)	0.709	**Perineal flap reconstruction type**			
**Cancer type**				IGAP	69 (84.1)	42 (37.5)	**< 0.001**
Locally advanced rectal cancer	55 (67.1)	43 (38.4)	**< 0.001**	SGAP	4 (4.9)	61 (54.5)	**< 0.001**
Recurrent rectal cancer	12 (14.6)	53 (47.3)	**< 0.001**	ALT	1 (1.2)	2 (1.8)	1.00
Anal SCC	3 (3.7)	5 (4.5)	1.00	VRAM	1 (1.2)	1 (0.9)	1.00
Recurrent anal SCC	3 (3.7)	6 (5.4)	0.736	Gracilis	6 (7.3)	7 (6.3)	0.779
Ileoanal pouch rectal cuff cancer	6 (7.3)	3 (2.7)	0.652	Medial thigh	3 (3.7)	6 (5.4)	0.736
Other*	3 (3.7)	2 (1.8)	0.171	Other**	1 (1.2)	4 (3.6)	0.399
**History of pelvic radiotherapy**				Two flap types combined	5 (6.1)	15 (13.4)	0.150
Yes	58 (70.7)	82 (73.5)	0.747	Vaginal reconstruction	27 (32.9)	21 (18.8)	**0.029**
No	24 (29.3)	30 (26.5)					

Abbreviations: ALT, Anterolateral Thigh; ELSiE, Extended Lateral Sidewall Excision; HTN, Hypertension; IBD, Inflammatory Bowel Disease; IGAP, Inferior Gluteal Artery Perforator; IHD, Ischaemic Heart Disease; SCC, Squamous Cell Carcinoma; SGAP, Superior Gluteal Artery Perforator; VRAM, Vertical Rectus Abdominis Musculocutaneous.

* 1re‐recurrent appendiceal cancer, 1 mucinous pelvic tumour on a background of Crohn′s disease, 1 malignant duplication cyst, 2 anal adenocarcinoma.

** Included 2 Singapore, 1 Deep inferior epigastric perforator, 2 rotational buttock flap.

Comparing patients undergoing conventional exenteration with those undergoing high complexity resections, there were no significant differences in age, sex, or major cardiovascular comorbidities (Table 1). Significantly more patients with inflammatory bowel disease and current smokers underwent conventional exenteration (*p* = 0.046). Overall, 84% of cases (n = 163) were performed for primary or locally recurrent rectal cancer, with a significantly higher proportion of recurrent disease requiring high complexity resection (*p* < 0.001).

Total pelvic exenteration (TPE) was required for half the patients; those undergoing high complexity resection were significantly more likely to undergo TPE than extended ELAPE (*p* < 0.001). The majority of high complexity resections (*n* = 80, 71.4%) involved both extended posterior and lateral resections incorporating both en bloc sacrectomy and ELSiE.

All resections were performed with curative intent. Gluteal artery perforator flaps were most frequently used for perineal reconstruction. SGAP flaps were much more frequently used in high complexity resections (54.5% vs. 4.9%, *p* < 0.001) due to available blood supply when performed with high sacrectomy and resection of internal iliac arteries below the superior gluteal artery origin. IGAP flaps were more common for conventional resections (84.1% vs. 37.5%, *p* < 0.001). A greater number of vaginal resections were performed in the conventional exenteration group, commensurate with a higher proportion of women in this cohort.

The median postoperative length of stay was 27 days (IQR 18‐49 days), significantly higher in the high complexity resection group (Table 2) (*p* < 0.001). The overall rate of major postoperative complications was 34.4%, with pelvic collection requiring drainage (11.3%) and urinary leak (9.7%) being the most frequent. Four postoperative deaths (2%) occurred within 30 days, all following a high complexity resection. Overall survival (OS) was reduced in the high complexity group with a 5‐year survival of 52% compared with a 78% 5‐year OS for patients in the conventional exenteration group (*p* = 0.015).

**Table 2:** Post operative outcomes.

	Conventional complex resection (*n* = 82)	High complexity resection (*n* = 112)	*p* value
Post operative length of stay, median days (IQR)	20.5 (13‐29.5)	32.5 (23‐57)	**<0.001**
Overall major complication	22 (26.8%)	48 (42.8%	**<0.001**
30 day mortality	0	4 (3.6)	0.139
**Perineal wound complications**			
**Early wound complication**			
Simple wound dehiscence requiring packing	9 (11.0)	9 (8.0)	0.618
Superficial dehiscence requiring VAC therapy	20 (24.3)	40 (35.7)	0.116
Full thickness dehiscence	2 (2.4)	1 (0.9)	0.572
Wound infection	19 (23.2)	7 (6.3)	**0.001**
Necrosis	3 (3.7)	7 (6.3)	0.524
**Major flap complications**			
Return to theatre for plastics revision within 90 days	4 (4.9)	10 (9.1)	0.402
Readmission with wound complication within 30 days	5 (6.1)	6 (5.4)	1.00
**Delayed/late wound complication**			
Sinus or fistula formation	5 (6.1)	12 (10.7)	0.312
Enteroperineal fistula	4 (4.9)	8 (7.1)	0.564
Symptomatic perineal hernia	5 (6.1)	3 (2.7)	0.286
Flap Revision	6 (7.3)	13 (11.6)	0.464

A wound complication of any type occurred in 104 patients (53.3%): 42 (40.4%) in the conventional resection group versus 62 (59.6%) in the high complexity group, *p* = 0.664 [Table 2]. The most frequent complication was superficial wound dehiscence requiring VAC therapy, occurring after 20 (24.3%) soft tissue resections and 40 (35.7%) high complexity resections, *p* = 0.116. The only wound‐related complication differing significantly between groups was wound infection requiring antibiotic therapy, occurring more commonly following conventional exenteration (23.2% vs. 6.3%, *p* = 0.001).

Ten patients (5.1%) developed partial flap necrosis requiring surgical debridement under general anaesthesia, with similar incidence between groups (6.3% vs. 3.7%, *p* = 0.524). Ten patients (9.1%) in the high complexity group returned to theatre for wound‐related issues within 90 days compared to 4 (4.9%) in the conventional group (*p* = 0.402). Eleven patients (5.6%) were readmitted within 30 days with wound‐related issues, with similar rates between groups (6.1% vs. 5.4%). There were no flap losses in the entire cohort.

The mean follow up was 26 months (IQR 9‐42months). Twelve patients (6%) were lost to follow up by 6 months post operatively. Seventeen patients (8.8%) developed chronic wound complications (persistent sinus or fistula), more frequently following high complexity resection (10.7% vs. 6.1%, *p* = 0.312). An underlying urine leak contributed to this in four cases. Enteroperineal fistula developed in four patients (4.9%) following conventional resection and eight (7.1%) following high complexity resection (*p* = 0.564). This complication significantly reduced mean overall survival: 42.3 months (95%CI 10.9–20.9) versus 74.0 months (95% CI 66.6–81.4), *p* = 0.023. Eight patients developed clinically significant perineal hernia, more commonly following conventional resection though not significantly (6.1% vs. 2.7%, *p* = 0.286).

### 3.1 Factors Associated With Perineal Wound Complications

On univariate analysis, age over 65 years, BMI greater than 30kg/m², type 2 diabetes, and TPE were associated with short‐term wound complications (Table 3). Only high BMI (HR 2.70, 95% CI 1.22–5.97, *p* = 0.014) and TPE (HR 2.13, 95% CI 1.07–4.23, *p* = 0.031) remained significant on multivariate analysis. Median length of stay was significantly increased following a short‐term wound complication: 30 days (IQR 22–59) versus 23 days (IQR 15–38), *p* = 0.001. [Table 3.]

**Table 3:** Risk factors for perineal wound complications.

	Univariate analysis		Multivariate analysis	
Risk Factors	OR (95%CI)	*p* value	OR (95%CI)	*p* value
**Early wound complication**				
Age > 65 years	2.13 (1.11–4.13)	0.023	1.73 (0.79–3.79)	0.164
Sex	1.28 (0.72–2.28)	0.464		
BMI > 30 kg/m^2^	2.95 (1.36–6.40)	0.008	2.70 (1.22–5.97)	0.014
Smoker	1.03 (0.32–3.32)	1.000		
History of RT	0.70 (0.38–1.32)	0.337		
Diabetes Mellitus	12.68 (1.60–100.27)	0.002	7.47 (0.88–63.07)	0.065
IHD	1.03 (0.25–4.25)	1.000		
High Complexity Resection	1.10 (0.62–1.95)	0.772		
TPE	1.87 (1.06–3.30)	0.044	2.13 (1.07–4.23)	0.031
Ureteric/ileal conduit leak	1.16 (0.45–3.00)	0.812		
**Late wound complication**				
Age > 65 years	1.27 (0.56‐2.90)	0.664		
Sex	1.17 (0.54‐2.55)	0.845		
BMI > 30kg/m^2^	1.39 (0.55–3.49)	0.469		
Smoker	0.43 (0.05–3.44)	0.695		
History of RT	0.86 (0.38–1.95)	0.677		
Diabetes Mellitus	2.65 (0.75–9.40)	0.124		
IHD	0.82 (0.77–0.88)	0.356		
Complex resection	1.49 (0.67–3.29)	0.434		
TPE	1.00 (0.47–2.14)	1.000		
Ureteric/ileal conduit leak	3.37 (1.21–9.34)	0.024		
**Readmission < 30days or reoperation < 90days**				
Age > 65 years	2.66 (1.07–6.6)	0.040		
Sex	1.46 (0.57–3.76)	0.495		
BMI > 30 kg/m^2^	1.27 (0.37–4.3)	0.746		
Smoker	0.88 (0.83–0.93)	0.367		
History of RT	1.83 (0.59–5.68)	0.448		
Diabetes Mellitus	1.63 (0.33–7.97)	0.630		
IHD	1.13 (0.13–9.64)	1.000		
Complex resection	1.07 (0.43–2.63)	1.000		
TPE	0.43 (0.17–1.10)	0.111		
Ureteric/ileal conduit leak	1.55 (0.41–5.81)	0.456		

The only significant factor associated with long‐term perineal wound issues was urine leak from either uretero‐ileal anastomosis or ileal conduit (HR 3.37, 95% CI 1.21‐9.34, *p* = 0.024). Age over 65 years was associated with readmission or reoperation within 90 days (HR 2.66, 95% CI 1.07‐6.6, *p* = 0.040).

Despite smoking being a known risk factor for perineal wound complications, no association with short or long‐term wound issues was observed in our cohort, though a few current smokers were included. The history of radiotherapy did not influence overall wound complication rates, although a few patients were not irradiated.

## 4 Discussion

This study reports one of the largest consecutive series of gluteal artery perforator flaps used for perineal reconstruction following complex rectal cancer surgery. We observed high overall morbidity, though predominantly from minor short‐term complications. Importantly, our consecutive series demonstrates that high complexity resections do not increase rates of perineal wound complications compared to conventional complex resections. This is a novel finding that contrasts with perceived wisdom that more extensive resections would lead to higher wound complication rates.

The increased overall complications and length of stay in the high complexity group are not unexpected, given the extent of resection and implications for patient mobility. However, it is reassuring that these resections do not confer greater perineal wound morbidity, supporting the use of these aggressive approaches for oncological benefit.

Recent systematic reviews have highlighted the substantial heterogeneity in reporting perineal reconstruction outcomes [17, 18]. Our study is unique in using the standardized ACPGBI/UKPEN Pelvic Exenteration Lexicon to define surgical complexity, enabling more meaningful comparisons with future studies [13]. Additionally, few large series have specifically examined gluteal fasciocutaneous perforator flaps, with most literature focusing on VRAM flaps [19].

Perineal wound complications prolong hospital stay, delay adjuvant therapies, and negatively impact treatment outcomes. A study from New Zealand found the mean cost of wound complications following abdominoperineal resection with primary perineal closure was $25,911 NZ (approximately £13,242) [9]. The risks are higher in complex cancer patients due to increased surgical morbidity (locally advanced tumours, extra‐levator dissection, prolonged operative time, hypothermia) and medical factors (preoperative radiotherapy, hypoalbuminemia, malnutrition) [20, 21, 22, 23].

Reported flap complication rates vary widely in the literature, from 9.5% to 55% [24, 25]. Our results align with these figures, with 36% of patients experiencing a major flap‐related complication. Obesity was the only potentially modifiable risk factor associated with increased short‐term wound complications in our cohort, consistent with previous studies of VRAM flap reconstruction [26, 27]. The finding that age over 65 years was associated with readmission/reoperation is important for preoperative risk counselling and discharge planning.

Recent meta‐analyses comparing flap reconstruction techniques have suggested potential advantages of gluteal flaps over VRAM for certain patients [18]. Our series confirms the reliability of gluteal fasciocutaneous perforator flaps even for extensive defects, with no complete flap losses observed.

The association between ureteric/ileal conduit leaks and long‐term wound complications is a significant finding that emphasizes the importance of multidisciplinary management. Meticulous urological technique and careful monitoring may help reduce these complications. The relationship between enteroperineal fistula and reduced overall survival is particularly concerning and warrants further investigation.

Growing interest in quality of life (QOL) outcomes following complex pelvic surgery makes our findings clinically relevant. Ramshorst et al. reported that patients with VRAM flap complications following pelvic exenteration had worse QOL regarding general health perceptions at 12 and 18 months postoperatively [26]. Future studies should investigate QOL outcomes specific to gluteal flap reconstruction.

Study limitations include its retrospective design, which precludes the determination of causation. The tertiary nature of our service means some patients are not locally based, potentially resulting in underreporting of complications managed by local teams. However, most patients received long‐term follow‐up through our service, likely capturing significant long‐term morbidity.

## 5 Conclusions

Gluteal fasciocutaneous perforator flaps provide reliable perineal reconstruction following complex rectal cancer resections, with no flap losses observed in this large consecutive series. Contrary to expectations, increasingly extensive resections resulting in larger perineal defects did not significantly increase perineal wound complication rates. BMI > 30kg/m² and total pelvic exenteration were significant predictors of short‐term wound complications, while age > 65 years was associated with readmission or reoperation. Further research should assess the impact of perineal wound complications on patients′ quality of life and explore strategies to mitigate these complications in high‐risk patients.

## Conflicts of Interest

The author declares no conflicts of interest.

## Data Availability

The data that support the findings of this study are available on request from the corresponding author. The data are not publicly available due to privacy or ethical restrictions.

## References

[1] M. E. Kelly , A. Aalbers , N. A. Aziz , et al., PelvEx Collaborative ., “Changing Outcomes Following Pelvic Exenteration for Locally Advanced and Recurrent Rectal Cancer,” BJS Open 3 (2019): 516–520. 31388644 10.1002/bjs5.50153 PMC6677093

[2] M. E. Kelly , R. Glynn , A. Aalbers , et al., PelvEx Collaborative ., “Factors Affecting Outcomes Following Pelvic Exenteration for Locally Recurrent Rectal Cancer,” British Journal of Surgery 105 (2018): 650–657. 29529336 10.1002/bjs.10734

[3] PelvEx Collaborative ., “Surgical and Survival Outcomes Following Pelvic Exenteration for Locally Advanced Primary Rectal Cancer: Results From an International Collaboration,” Annals of Surgery 269 (2019): 315–321. 28938268 10.1097/SLA.0000000000002528

[4] T. Milne , M. J. Solomon , P. Lee , J. M. Young , P. Stalley , and J. D. Harrison , “Assessing the Impact of a Sacral Resection on Morbidity and Survival After Extended Radical Surgery for Locally Recurrent Rectal Cancer,” Annals of Surgery 258 (2013): 1007–1013. 23364701 10.1097/SLA.0b013e318283a5b6

[5] M. Mughal , R. Baker , A. Muneer , and A. Mosahebi , “Reconstruction of Perineal Defects,” Annals of The Royal College of Surgeons of England 95 (2013): 539–544. 24165333 10.1308/003588413X13629960047155 PMC4311526

[6] D. Y. S. Witte , G. H. van Ramshorst , O. Lapid , M. B. Bouman , and J. B. Tuynman , “Flap Reconstruction of Perineal Defects After Pelvic Exenteration: A Systematic Description of Four Choices of Surgical Reconstruction Methods,” Plastic & Reconstructive Surgery 147 (2021): 1420–1435. 33973948 10.1097/PRS.0000000000007976

[7] C. Devulapalli , A. T. Jia Wei , J. R. DiBiagio , et al., “Primary Versus Flap Closure of Perineal Defects Following Oncologic Resection,” Plastic & Reconstructive Surgery 137 (2016): 1602–1613. 26796372 10.1097/PRS.0000000000002107

[8] D. C. Moraru and V. Scripcariu , “Decisional Algorithms for the Reconstruction of Pelviperineal Defects After Total Pelvic Exenteration: A Review,” Revista Medico 122 (2018): 40–50.

[9] J. Woodfield , M. Hulme‐Moir , and J. Ly , “A Comparison of the Cost of Primary Closure or Rectus Abdominis Myocutaneous Flap Closure of the Perineum After Abdominoperineal Excision,” Colorectal Disease 19 (2017): 934–941. 28436214 10.1111/codi.13690

[10] I. Shaikh , W. Aston , G. Hellawell , et al., “Extended Lateral Pelvic Sidewall Excision (ELSiE): An Approach to Optimize Complete Resection Rates in Locally Advanced or Recurrent Anorectal Cancer Involving the Pelvic Sidewall,” Techniques in Coloproctology 18 (2014): 1161–1168. 25380742 10.1007/s10151-014-1234-9

[11] I. Shaikh , I. Holloway , W. Aston , et al., “High Subcortical Sacrectomy: A Novel Approach to Facilitate Complete Resection of Locally Advanced and Recurrent Rectal Cancer With High (S1‐S2) Sacral Extension,” Colorectal Disease 18 (2016): 386–392. 26638828 10.1111/codi.13226

[12] I. Drami , J. A. Fletcher , A. Corr , et al., “Total Pelvic Exenteration With “High and Wide” Sacrectomy for Recurrent Rectal Cancer: A Video Vignette,” Colorectal Disease 24 (2022): 1625–1626. 35730692 10.1111/codi.16230

[13] N. J. Hodges , A. Dinnewitzer , R. Hompes , et al., “A Consensus Terminology for Description and Documentation of Extensive Pelvic Exenteration for Locally Advanced and Recurrent Rectal Cancer,” Colorectal disease: the official journal of the Association of Coloproctology of Great Britain and Ireland 25 (2023): 1022–1032.

[14] D. Dindo , N. Demartines , and P. A. Clavien , “Classification of Surgical Complications: A New Proposal With Evaluation in a Cohort of 6336 Patients and Results of a Survey,” Annals of Surgery 240 (2004): 205–213. 15273542 10.1097/01.sla.0000133083.54934.ae PMC1360123

[15] A. Hainsworth , M. al Akash , P. Roblin , P. Mohanna , D. Ross , and M. L. George , “Perineal Reconstruction After Abdominoperineal Excision Using Inferior Gluteal Artery Perforator Flaps,” British Journal of Surgery 99 (2012): 584–588. 22231559 10.1002/bjs.7822

[16] J. A. Fletcher , I. Drami , M. A. West , et al., “Inferior Gluteal Artery Perforator Flap Reconstruction of the Perineum: A Video Vignette,” Colorectal Disease 24 (2022): 1098. 35426969 10.1111/codi.16152

[17] E. P. Colov , M. Klein , and I. Gögenur , “Wound Complications and Perineal Pain After Extralevator Versus Standard Abdominoperineal Excision: A Nationwide Study,” Diseases of the Colon & Rectum 59 (2016): 813–821. 27505109 10.1097/DCR.0000000000000639

[18] H. Bouman , A. D. S. Wilk , M. A. Kaijser , et al., “Reconstructive Options Following Abdominoperineal Resection for Anorectal Malignancies: A Literature Review,” World Journal of Surgical Oncology 21 (2023): 38.36747272

[19] S. S. Qiu , M. Jurado , and B. Hontanilla , “Comparison of TRAM Versus Diep Flap in Total Vaginal Reconstruction After Pelvic Exenteration,” Plastic and Reconstructive Surgery 132 (2013): 1020e–1027e. 10.1097/PRS.0b013e3182a97ea2 24281607

[20] G. D. Musters , D. A. M. Sloothaak , S. Roodbeen , A. A. W. van Geloven , W. A. Bemelman , and P. J. Tanis , “Perineal Wound Healing After Abdominoperineal Resection for Rectal Cancer: A Two‐Centre Experience in the Era of Intensified Oncological Treatment,” International Journal of Colorectal Disease 29 (2014): 1151–1157. 25064389 10.1007/s00384-014-1967-y

[21] K. M. Bullard , J. L. Trudel , N. N. Baxter , and D. A. Rothenberger , “Primary Perineal Wound Closure After Preoperative Radiotherapy and Abdominoperineal Resection Has a High Incidence of Wound Failure,” Diseases of the Colon & Rectum 48 (2005): 438–443. 15719190 10.1007/s10350-004-0827-1

[22] C. K. Christian , M. R. Kwaan , R. A. Betensky , E. M. Breen , M. J. Zinner , and R. Bleday , “Risk Factors for Perineal Wound Complications Following Abdominoperineal Resection,” Diseases of the Colon & Rectum 48 (2005): 43–48. 15690656 10.1007/s10350-004-0855-x

[23] A. A. Althumairi , J. K. Canner , S. L. Gearhart , B. Safar , J. Sacks , and J. E. Efron , “Predictors of Perineal Wound Complications and Prolonged Time to Perineal Wound Healing After Abdominoperineal Resection,” World Journal of Surgery 40 (2016): 1755–1762. 26908238 10.1007/s00268-016-3450-0

[24] X. Yang , M. Wei , X. Yang , et al., “Primary vs. Myocutaneous Flap Closure of Perineal Defects Following Abdominoperineal Resection for Colorectal Disease: A Systematic Review and Meta‐Analysis,” Colorectal Disease 21 (2019): 138–155. 30428157 10.1111/codi.14471

[25] M. S. Johnstone , “Vertical Rectus Abdominis Myocutaneous Versus Alternative Flaps for Perineal Repair After Abdominoperineal Excision of the Rectum in the Era of Laparoscopic Surgery,” Annals of Plastic Surgery 79 (2017): 101–106. 28542071 10.1097/SAP.0000000000001137

[26] G. H. van Ramshorst , J. M. Young , and M. J. Solomon , “Complications and Impact on Quality of Life of Vertical Rectus Abdominis Myocutaneous Flaps for Reconstruction in Pelvic Exenteration Surgery,” Diseases of the Colon & Rectum 63 (2020): 1225–1233. 33216493 10.1097/DCR.0000000000001632

[27] R. A. Nelson and C. E. Butler , “Surgical Outcomes of Vram Versus Thigh Flaps for Immediate Reconstruction of Pelvic and Perineal Cancer Resection Defects,” Plastic & Reconstructive Surgery 123 (2009): 175–183. 19116551 10.1097/PRS.0b013e3181904df7

